# Periodic breathing in healthy young adults in normobaric hypoxia equivalent to 3500 m, 4500 m, and 5500 m altitude

**DOI:** 10.1007/s11325-019-01829-z

**Published:** 2019-04-10

**Authors:** Stephan Pramsohler, Robert Schilz, Andreas Patzak, Linda Rausch, Nikolaus C. Netzer

**Affiliations:** 1 Dept. of Psychology and Sports Science, Hermann Buhl Institute for Hypoxia and Sleep Medicine Research, University of Innsbruck, Ghersburgstr. 9, 83043 Bad Aibling, Germany; 2 University Hospitals of Cleveland and Case University School of Medicine, 11100 Euclid Avenue, Cleveland, OH 44106 USA; 3 Charité-Universitätsmedizin Berlin, Institute for Vegetative Physiology, Chariteplatz 1, 10117 Berlin, Germany; 4 Dept. of Psychology and Sport Science, University Innsbruck, Fürstenweg 185, 6020 Innsbruck, Austria; 5 Division of Sports Medicine and Rehabilitation, Department of Medicine, University Ulm, Leimgrubenweg 14, 89070 Ulm, Germany

**Keywords:** Hypoxia, Periodic breathing, Sleep, Polysomnography, Altitude

## Abstract

**Purpose:**

The occurrence of periodic breathing (PB) at high altitude during sleep and the quality of sleep are individually different and influenced by multiple factors including sex. Although poor sleep quality at high altitude might not be directly linked to oxygen desaturations, the PB upsurge at high altitude leads to significant oscillations in oxygen saturation.

**Methods:**

Thirty-three students were recruited. Participants were randomly assigned to three groups (A, B, C) sleeping one full night in a dormitory with normobaric hypoxia at a F_I_O2 of 14.29% (A), a F_I_O2 of 12.47% (B), or a F_I_O2 of 10.82% (C). Full polysomnography was performed in each participant.

**Results:**

Mean total sleeping time decreased significantly with increasing hypoxia (*p* < 0.001). Respiratory events changed from central hypopneas to central apneas (CA) with increasing hypoxia: CA = 17.8%, 50.0%, 92.2% of AHI (37.96 events per hour (*n*/h), 68.55 *n*/h, 93.44 *n*/h). AHI (*p* = 0.014) and time duration of respiratory events (*p* = 0.003) were significantly different between sexes, both greater in men. REM sleep was reduced.

**Conclusions:**

Men tend to be more prone to PB in normobaric hypoxia. Further research should implicate a longer acclimatization period around simulated 4500 m in order to find out if the exponential increase in PB between 4500 m and 5500 m could be shifted to lower hypoxic levels, i.e., higher altitudes.

## Introduction

Periodic breathing (PB) during sleep has shown to occur in almost every individual reaching a certain altitude, depending on its genetics, training status, sex, previous acclimatization, and preexisting diseases and medication [1]. The mechanisms leading to PB in hypoxia are acceptably explored [2]. Although, certain questions remain unanswered as, at which altitude does it occur or how are sex and other factors influencing PB [3, 4]. PB at altitude is caused by respiratory instability due to a disbalance of chemical stimuli [5]. The time lag between peak ventilation and peak oxygen saturation is increased at altitude compared to sea level causing a late start of the corrective response and sleeping patterns become severely disturbed [6]. The nightly desaturations accompanying the central apneas and poor sleep due to arousals at altitude have shown to play an important role in the development of acute mountain sickness (AMS) [7]. However, individual incidence in different altitudes or the occurrence of a specific upsurge of PB in normobaric hypoxia has not been sufficiently explored. For all we know from studies in hypobaric hypoxia, PB increases linear with increasing altitude and occurs rather individually [8]. Since sex differences can affect the symptoms and the occurrence of sleep apnea, they are of special interest when it comes to altitude sleep [9, 10]. In hypobaric environments, the increased hypoxic chemo-instability in men seems to be driving increased PB at altitude [11–13]. This sex difference has also been reported to be based on hormonal mechanisms directly and indirectly contributing to ventilatory control and central breathing stimulation in subjects at altitude, however, these data are still controversially discussed [8–14]. The understanding of PB in normobaric hypoxia in different simulated altitudes could add to a better understanding of the underlying mechanisms and sex-related differences to hypoxia. Therefore, our aim was to study differences in PB in normobaric hypoxia equivalent to 3500 m, 4500 m, and 5500 m (F_I_O2 = 14.29%, 12.47%, 10.82%). We hypothesized that there might be an inspired oxygen fraction (F_I_O_2_)-dependent upsurge for PB and that there are sex-related differences in the occurrence and degree of PB.

## Materials and methods

### Subjects

Thirty-three healthy students have been recruited from the University of Ulm and the University of Innsbruck and gave written informed consent. Inclusion criteria were the absence of pre-diagnosed sleeping disorders and an overall good health status as non-smoker. All students were questioned a priori by a physician to assure health status and exclude pregnancy. According to the physician, none of the students was overweight indicating a BMI between 18.5 and 24.9 kg/m^2^. All students were of German or Austrian ethnicity. Preexisting sleep disorders have been excluded via BERLIN questionnaire. We evaluated 19 male and 14 female subjects with a mean age of 23.36 ± 2.52 years (Table 1). All participants were randomly assigned into three groups. Group A contained 5 male and 6 female subjects (age 24.45 ± 1.75 years), group B 8 male and 3 female subjects (age 24.64 ± 1.91 years), and group C 6 male and 5 female subjects (age 21.00 ± 2.10 years) (Table 1). They had comparable daytime routines since they followed the same course program containing lectures and physical activity for the past semester as well as hiking at altitudes < 2000 m the days before the measurements. Previous exposition to moderate and high altitudes during the last 2 weeks prior to study start could be excluded for all subjects. Group characteristics are displayed in Table 1.

**Table 1:** Group characteristics. Values are presented as means ± SD

	F_I_O_2_ (%)	Simulated altitude _(m)_	*n*	Sex (m/f)	Age (years)
Group A	14.29	3500	11	5/6	24.45 (± 1.75)
Group B	12.47	4500	11	8/3	24.64 (± 1.91)
Group C	10.82	5500	11	6/5	21.00 (± 2.10)

F_I_O_2_, inspired oxygen fraction

### Procedure

The three groups were assigned to three different normobaric altitude simulations. Group A at a F_I_O2 of 14.29%, Group B at a simulated F_I_O2 of 12.47% and Group C at a F_I_O2 of 10.82%. According to the assigned group, the corresponding altitudes were Group A at 3500 m, Group B at 4500 m, and Group C at 5500 m. Group B and C underwent an acclimatization night at a F_I_O2 of 14.29% (equivalent to 3500 m) the night before the actual testing night in order to prevent symptoms of acute mountain sickness (AMS). The trial took place in the normobaric altitude sleeping room of the Hermann Buhl Institute for Hypoxia and Sleep Medicine Research. Normobaric hypoxia was provoked by an oxygen expulsion System (normobaric hypoxia, low oxygen systems; Berlin-Buch, Germany). This allows reducing oxygen in the whole chamber down to a minimum of 9.3%. The participants’ medical history was assessed by an experienced physician. Two students at a time were connected to a 12-channel PSG each study day (Sidas, Stimotron Inc., Roth, Germany). Polysomnography was carried out and scored by a sleep physician according to the American Academy of Sleep Medicine Standard of 2017 [15]. Monitoring time was 11:00 pm until 06:30 am. During this period, following data was collected continuously: heart rate (HR), EMG, EEG, EKG, EOG, peripheral oxygen saturation (SpO2), nasal air flow (NAF), position, and abdominal and thoracic movement. The assessment of AMS symptoms was self-administered by each participant upon awakening using the Lake Louise Score. If subjects experienced symptoms of AMS higher than 6 on the Lake Louise Score, they were allowed to interrupt measurements and leave the hypoxic room.

### Statistical analysis

Data are presented as means ± standard deviation (SD). Data analyses were performed with the SPSS statistical software package (PASW Statistics for Windows version 21.0, SPSS Inc., Chicago, IL, USA). Normal distribution of data has been tested via the Shapiro-Wilk test and has been visually checked. A multifactor ANOVA was applied to identify differences between altitudes and sex. Significance level was set at *p* < 0.05. Post-hoc power calculation via G-power for sex differences in the main parameter AHI gave a power of 0.97.

**Fig. 1:**
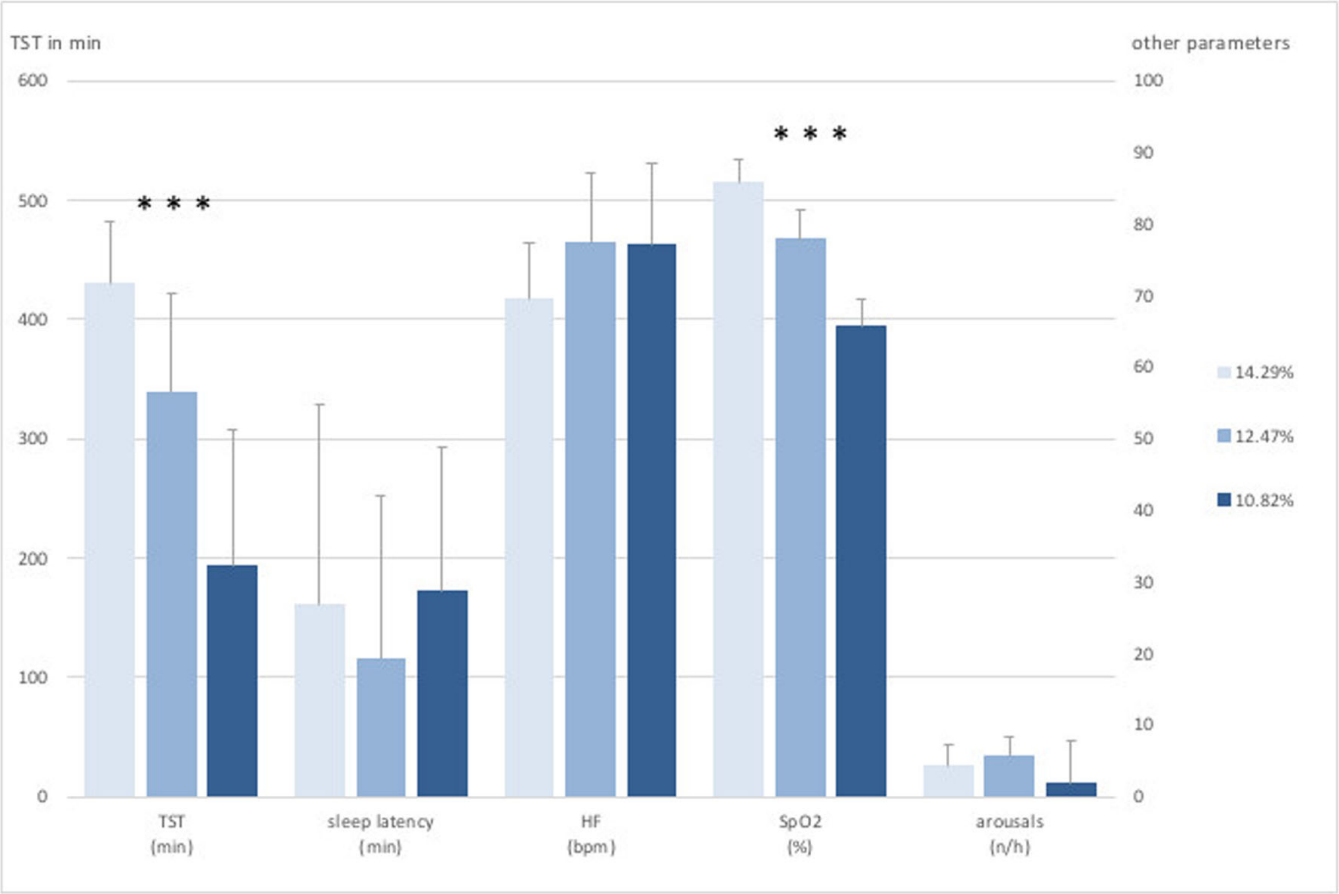
General polysomnographic data recorded at 3 different levels of normobaric hypoxia at an F_I_O_2_ of 14.29% (3500 m), 12.47% (4500 m), and 10.82% (5500 m). Values are presented as means ± SD. Legend: TST, total sleeping time, HF, heart frequency, SpO2, peripheral oxygen saturation; arousals: events per hour (*n*/h) * = level of significance, *p* ≤ 0.001 (in regard to different hypoxic conditions)

## Results

**Fig. 2:**
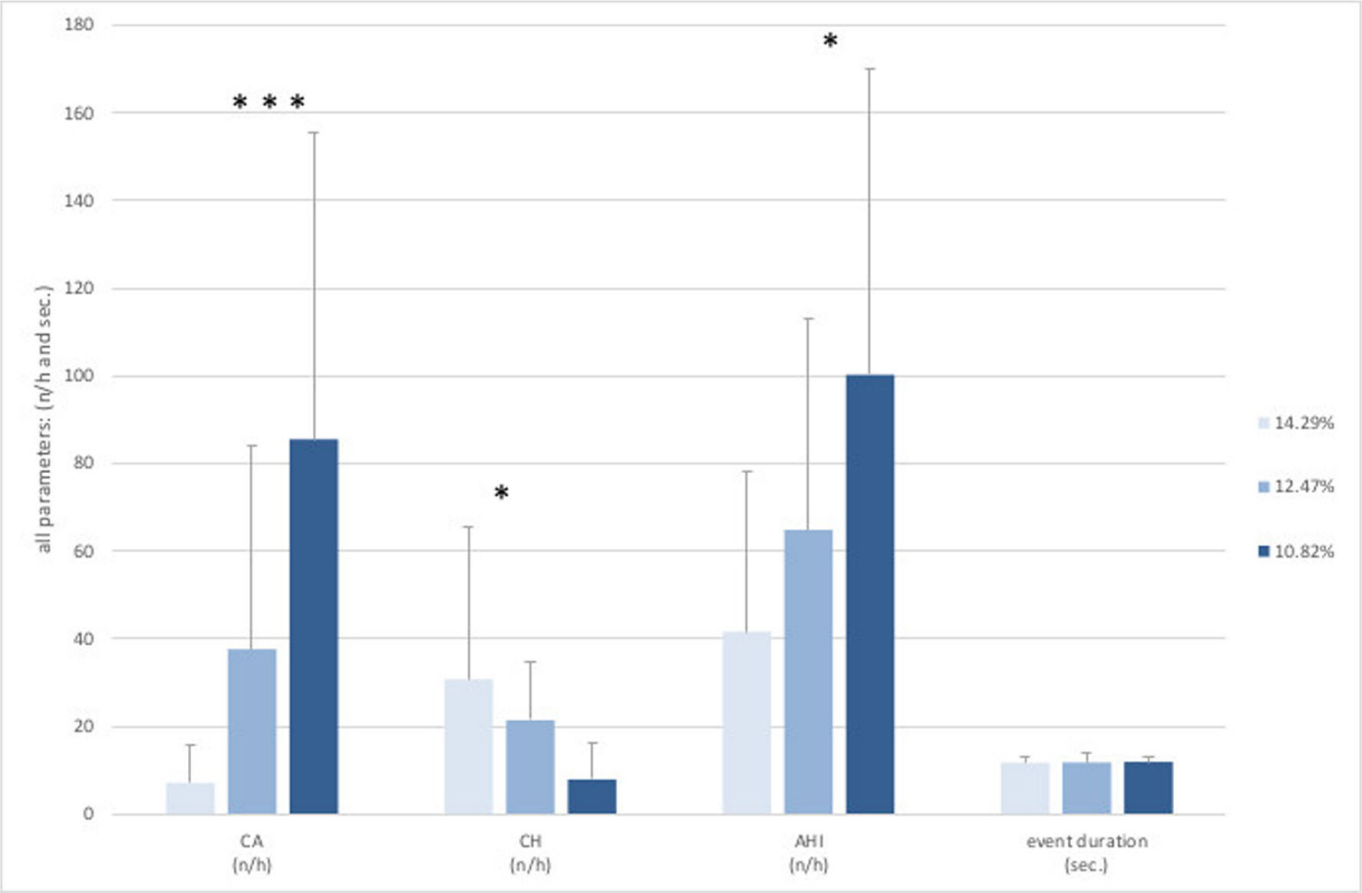
Respiratory parameters from polysomnographic data recorded at 3 different levels of normobaric hypoxia at an F_I_O_2_ of 14.29% (3500 m), 12.47% (4500 m), and 10.82% (5500 m). Values are presented as means ± SD. CA, central apneas; CH, central hypopneas; AHI, apnea hypopnea index; *n*/h = events per hour; levels of significance, * = *p* ≤ 0.05; *** = *p* ≤ 0.001(in regard to different hypoxic conditions)

**Fig. 3:**
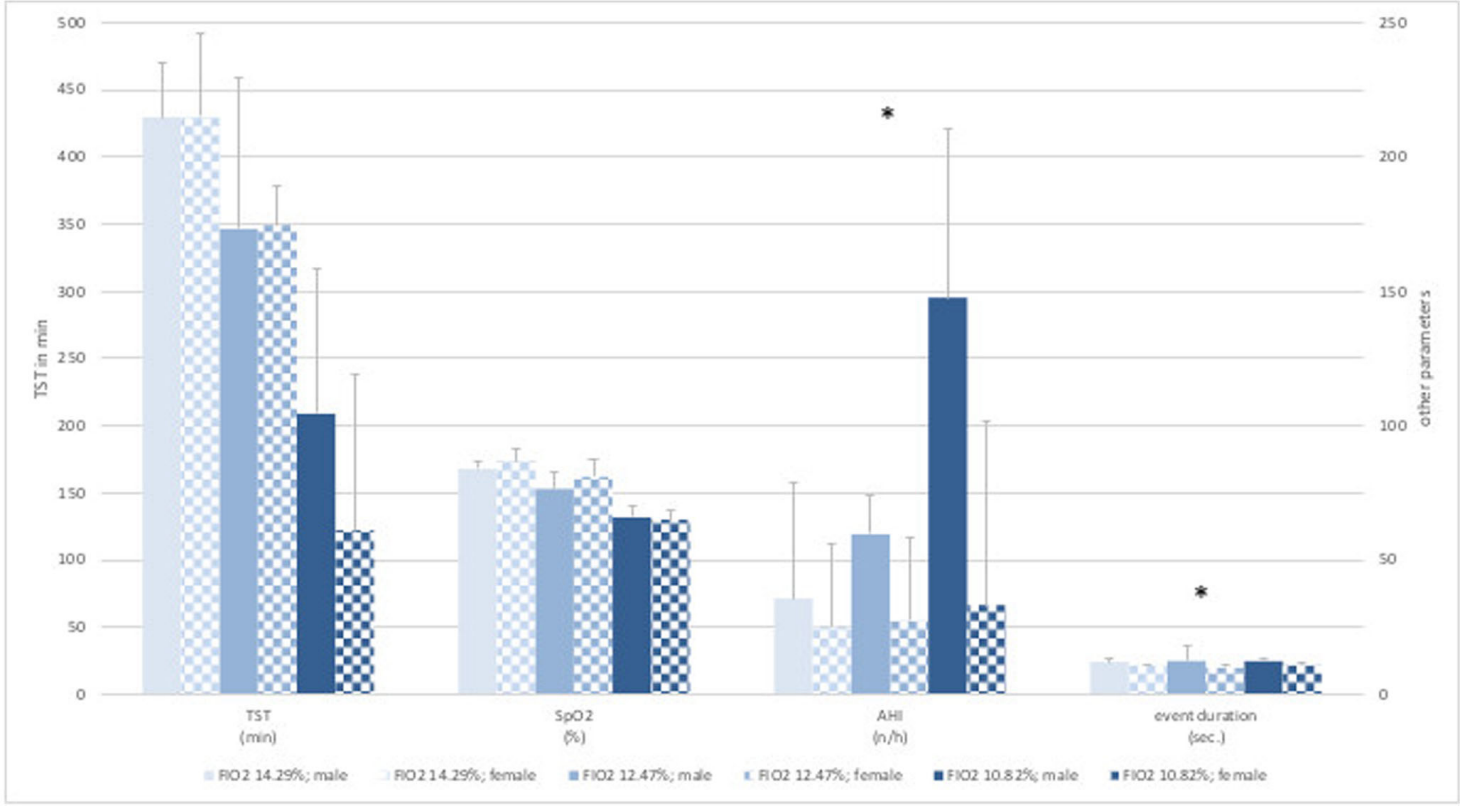
Sex differences. Polysomnographic data recorded at 3 different levels of normobaric hypoxia at an F_I_O_2_ of 14.29% (3500 m), 12.47% (4500 m), and 10.82% (5500 m). Values are presented as means ± SD. Legend: F_I_O2, inspired oxygen fraction, SpO2, peripheral oxygen saturation, TST, total sleeping time, AHI, apnea hypopnea index (*n*/h = events per hour); levels of significance between sexes: SpO2, *p* = 0.400; TST, *p* = 0.781; AHI, *p* = 0.014; event duration, *p* = 0.003

The measurements and altitude simulations at a F_I_O_2_ of 14.29% (equivalent to 3500 m) and 12.47% (equivalent to 4500 m) were well tolerated. None of the participants had to leave the hypoxic room due to moderate AMS symptoms (Lake Louise Score > 6). At the measurements at a F_I_O_2_ of 10.82% (equivalent to 5500 m) almost all participants were suffering from moderate AMS (Lake Louise Score > 6) symptoms and left the hypoxic room after 4 to 7 h. We observed a highly significant decrease in total sleeping time (*p* < 0.001) and peripheral oxygen saturation (*p* < 0.001) with decreasing F_I_O_2_ considering all subjects (Table 2). The heart rate was slightly increased with greater simulated altitudes but showed no significance considering all subjects (*p* = 0.100) (Fig. 1). We could measure a significant shift from mostly hypopneas towards apneas at a F_I_O2 of 12.47% (4500 m) and at a F_I_O2 of 10.82% (5500 m) (*p* = 0.001, *p* = 0.032) with an increase of total respiratory events, but found no changes in duration of the events (*p* = 0.527). There was a significant increase in mean AHI (*p* = 0.017). (Fig. 2) The arousal frequency did not change from a F_I_O2 of 14.29% (3500 m) up to a F_I_O2 of 12.47% (4500 m) in all participants and at a F_I_O2 of 10.82% (5500 m) only one subject expressed arousals (87/h) by the classic definition of short (few seconds) alpha rhythm in the EEG (Fig. 1). All other subjects had no arousals during sleep phases but only full awakenings from respiratory disturbances. REM sleep was low, compared to normal, at all hypoxia levels (7.45%, 10.8%, 5.8% of TST) with no difference between sexes [16]. Significant sex differences were seen in AHI and event duration parameters. Female subjects showed a lower AHI (*p* = 0.014) as well as a shorter event duration (*p* = 0.003) in all hypoxic conditions. No significant sex differences could be seen in SpO_2_ or TST during all hypoxic conditions (Fig. 3).

**Table 2:** Distribution of non-REM and REM sleep in different hypoxic conditions and display of SpO_2_ characteristics in regard to total sleeping time (TST). Values are presented as means ± SD

F_I_O_2 (%)_	TST _(min)_	nREM_(%)_	REM_(%)_	SpO_2(%) minimum_	SpO_2 (below 90%)_	SpO2 _(below 80%)_
14.29	430 (± 51.56)	92.91 (± 5.61)	7.36 (± 6.07)	66.0 (± 10.76)	79.91 (± 28.70)	6.64 (± 13.77)
12.47	340 (± 83.28)	90.36 (± 9.30)	10.82 (± 9.24)	56.82 (± 9.81)	98.27 (± 2.24)	58.0 (± 30.90)
10.82	188 (± 118.20)	98.41 (± 13.88)	5.96 (± 5.64)	55.60 (± 4.03)	99.73 (± 0.85)	97.76 (± 4.07)

F_I_O_2_, inspired oxygen fraction in %; TST, total sleep time in minutes; nREM/REM, (non) rapid eye movement sleep in % of TST; SpO_2_, oxygen saturation values in % of TST

## Discussion

To our knowledge, this is the first study to assess PB in normobaric hypoxia at different simulated altitudes. The low impact of disruptive factors using normobaric hypoxia compared to hypobaric chambers like in the Operation Everest II in Loma Linda (California) or real altitude seems to be unique [17]. Due to our findings, the occurrence of PB increases with decreasing F_I_O_2_ first in a more linear matter and then with an exponential upsurge between a F_I_O2 of 12.47% (4500 m) and a F_I_O2 of 10.82% (5500 m). Due to the signal chain of the carotidal chemoreceptors and the delay of the feedback response, PB patterns are more pronounced in hypoxic environments in an hypoxic environment of a F_I_O2 of 10.82% (5500 m). This applies more to non-acclimatized subjects. PB has been thought by some colleagues to have a stabilizing effect on oxygen saturation and to be an acute adaption to the demanding situation of hypoxia [5, 18–20]. Our data supports this theory since PB is increased reaching critical altitudes. However, in our sample, we could not establish a correlation between AHI and SpO_2_. Considering the prevalence of males in the hypoxic condition with an F_I_O2 of 12.47% (4500 m) we cannot exclude an effect on the mean AHI, given the small sample size. However, we report a homogenous subject group concerning age, as the age difference was not significant between groups. Therefore, the anthropometric age data should have not influenced measured parameters.

TST was significantly reduced with greater simulated altitudes. The disturbance due to the lower F_I_O_2_ seems to impact sleep severely and does not allow longer REM periods [21, 22]. If this is due to the low oxygen levels alone or co-affected by the mechanical disturbance due to PB remains to be investigated [21]. Surprisingly in our experiment and in opposition to previous studies like Operation Everest II, arousals did not play a predominant role [17]. Although there could be observed a slight increase in arousal frequency from a F_I_O2 of 14.29% (3500 m) up to a F_I_O2 of 12.47% (4500 m), subjects either awoke or slept without arousals at a F_I_O2 of 10.82% (5500 m) with few arousals in their short sleep periods. This opposing finding could be due to less external disruptive factors in the normobaric hypoxia room, where sleep was not disturbed by compressors. More studies on this matter are needed.

Consistent with other studies, the shift from mostly hypopneas to apneas was quite significant. The lower oxygen levels seem to amplify respiratory responses and lead to a higher oscillation. The duration of respiratory events did not seem to be affected by lower F_I_O_2_ levels. According to Orr et.al. 2017, the duration of high altitude provoked PB events remains quite stable at approximately 10 s [23]. PB was more pronounced in male subjects which could indicate higher instability of the carotidal chemoreceptors in men [24]. Furthermore, male subjects seem to show a longer event duration regardless to the dose of hypoxia compared to women. This could be due to higher lung volumes and the slower breathing frequency male subjects show in general and therefore a slower responsiveness to carotidal signals [25]. The sex difference could also be related to the effect of sex hormones directly and indirectly affecting respiration and ventilation mechanisms as well as cerebral blood flow regulations [14]. Hormones such as estrogens and androgens influence cerebral blood circulation, which in turn affects central chemoreflex activity [26]. During normal menstrual cycle, estrogens, androgens, and testosterones take action in the central neural control of breathing, which affects cyclic fluctuations in ventilation. The effect of cerebral blood flow exerted by female hormones might contribute to improve the stability of ventilator control [27]. Additional information on the menstrual cycle phase of our female subjects as well as application of contraceptives could have supported this theory and should be assessed in further investigations. Usually, very few obstructive events are registered in healthy subjects at sea level, however, we could not detect any obstructive events in our subjects at altitude. We assume that at the studied altitudes, the frequent periodic breathing might have masked few obstructive events.

One main limitation of this study is the lack of baseline polysomnography at sea level which would have provided us with individual starting points regarding respiratory parameters. We tried to address this shortcoming with a detailed assessment of the subjects’ medical history excluding any cardiorespiratory conditions, which might influence respiration at sea level and non-hypoxia induced PB-related events at altitude. Although the number of incidences of preexisting sleep disorders in young subjects is rising, we assumed that the prevalence of preexisting sleep disorders in adults of age is still higher [28]. Therefore, we would anticipate a rather low incidence of preexisting sleep disorders in our study population. Due to the fact, that we aimed at the assessment of acute hypoxia exposure, we did not have a familiarization period or acclimatization nights at conditions of a F_I_O2 of 14.29% (3500 m) and a F_I_O2 of 12.47% (4500 m). Furthermore, some subjects interrupted sleep at a F_I_O2 of 10.82% (5500 m) because of AMS symptoms. This might have led to shorter TST.

In conclusion, our findings indicate that from a F_I_O2 of 12.47% (4500 m) up to a F_I_O2 of 10.82% (5500 m) PB increases exponentially. This is of importance to know for mountaineers and other persons reaching very high altitudes for recreational or professional purposes because the exponential increase of periodic breathing might have significant impact on health and wellbeing. It is very likely that PB has a SpO_2_ stabilizing effect and is a necessary adaptive response to hypoxia. Considering the fact, that men tend to be more prone to PB than women in normobaric hypoxia, we assume that female sex hormones regulating the menstrual cycle also contribute to improve nightly ventilator control stability in hypoxia. Further investigations examining the hormonal threshold during sleep while exposed to hypoxia concerning PB are suggested. Overall, possible differences of normobaric (NH) and hypobaric (HH) hypoxia regarding ventilator response during sleep have to be taken into account [29]. HH could induce lower nocturnal oxygen saturation values and more AHI compared to NH [19]. The main difference could lie in NO metabolism altering pulmonary capillary vasodilation or an increase of physiological dead space [30, 31]. These hypotheses will need to be confirmed in further studies. However, the occurrence of PB appears to show similar metrics in both, NH and HH, which still leaves normobaric hypoxia a valuable tool for further investigations [19].

Further research should implicate a longer acclimatization period around a F_I_O2 of 12.47% (4500 m) in order to find out if the exponential increase in PB between a F_I_O2 of 12.47% (4500 m) and a F_I_O2 of 10.82% (5500 m) could be shifted to lower hypoxic levels, i.e.,higher altitudes.
